# Proteins Involved in Synaptic Plasticity Are Downregulated in the Cerebrospinal Fluid of Infants With Clinical Sepsis Complicated by Neuroinflammation

**DOI:** 10.3389/fncel.2022.887212

**Published:** 2022-05-11

**Authors:** Ping-Ping Jiang, Shan-Shan Peng, Stanislava Pankratova, Ping Luo, Ping Zhou, You Chen

**Affiliations:** ^1^Department of Veterinary and Animal Sciences, University of Copenhagen, Copenhagen, Denmark; ^2^School of Public Health, Sun Yat-sen University, Guangzhou, China; ^3^Department of Neonatology, Bao'an Women and Children's Hospital, Jinan University, Shenzhen, China

**Keywords:** neonatal sepsis, neuroinflammation, CSF, proteomics, amyloid-β precursor protein (APP)

## Abstract

Newborn infants are prone to sepsis and related inflammation of different organs. Neuroinflammation has been associated with long-term adverse neuronal (neuropsychiatric/neurodegenerative) outcomes, including attention deficit hyperactivity disorder (ADHD) or even Alzheimer's disease. Despite a vast number of findings on sepsis-induced inflammatory responses in the central nervous system (CNS), how neuroinflammation affects brain development remains largely elusive. In this study, neonates with clinical sepsis and screened for meningitis were included and classified by the neuroinflammation status based on cerebrospinal fluid (CSF) parameters (INF vs. NOINF). CSF samples collected from clinical screening were subjected to proteomics analysis. Proteins with differential abundance were subjected to enrichment analysis to reveal affected biological pathways. INF and NOINF infants had similar demographic data and hematological and biochemical parameters in blood and CSF. The CSF proteomes were essentially different between the two groups. All 65 proteins with differential abundance showed lower abundance in the INF group and functionally covered pivotal developmental processes, including axonal and synaptic function and extracellular homeostasis. CSF proteins, PTPRZ1 and IGFBP4, were correlated with C-reactive protein (CRP) and ratios of immature/total neutrophils in blood. In general, a substantial change in the CSF protein profile was found under neuroinflammation, and these changes are related to systemic conditions. The results suggest that changes in CSF proteins may be involved in sepsis-affected neurodevelopment, such as disturbances in circuit formation, which has the potential to predispose neonates to long-term adverse outcomes.

## Introduction

Neonatal sepsis is associated with 10–30% mortality in infants (Schlapbach et al., 2011) and with acute morbidities of the central nervous system (CNS), including bacterial meningitis, white matter injury and impairment in mental and motor development (van Vliet et al., 2013), as has been shown in humans and animal models (Du et al., 2011; Mottahedin et al., 2017; Hsieh et al., 2018). The neonatal period is a critical time for CNS development, with active axonal arborization, synaptogenesis, myelination and gliogenesis (Katz and Shatz, 1996; Mottahedin et al., 2017). CNS exposure to proinflammatory stimuli during this period may predispose to neurological and psychiatric disorders in adolescence and adulthood, such as autism spectrum disorders (ASD), schizophrenia, multiple sclerosis, and cognitive impairment (Van Steenwinckel et al., 2014; Mottahedin et al., 2017). In addition to being one of the leading contributors to CNS injury, neuroinflammation also sensitizes the developing brain to later hypoxia-ischemia insult (Maj et al., 2004). Thus far, the immune and inflammatory responses in the CNS have been well-investigated in adults (Strunk et al., 2014; Kölliker-Frers et al., 2021), with activation of pattern recognition receptors (PRRs), involved cells and mediating cytokines and chemokines identified (Hagberg et al., 2012). However, few studies have focused on the neurofunctional pathways affected by neuroinflammation in the neonatal CNS.

Cerebrospinal fluid (CSF), due to its proximity and direct interaction with the brain interstitial fluid, can accurately reflect the pathological changes in the CNS and thus is an ideal source for assessing the biochemical changes related to neurological disorders (Gaetani et al., 2020). A number of CSF proteins have been identified as biomarkers of different CNS diseases, such as Alzheimer's disease (AD) (Johnson et al., 2020), dementia (Abu-Rumeileh et al., 2020), multiple sclerosis (McKay et al., 2021) and septic encephalopathy (Hamed et al., 2009). We hypothesized that CSF proteins may provide information on the developmental impairment of the CNS under neuroinflammation in neonatal sepsis. In this study CSF samples from infants with diagnosed clinical sepsis who were screened for meningitis were collected, and the CSF proteome was compared between the groups with and without neuroinflammation. The data revealed that sepsis-induced neuroinflammation is associated with decreased levels of proteins that play essential roles in synaptic homeostasis and plasticity, which in turn may impact the development of CNS impairment among newborn preterm infants.

## Materials and Methods

### Study Population and Clinical Information

The sample collection was conducted at the Department of Neonatology of Bao'an Women and Children's Hospital, Shenzhen, China. Ethical approval was obtained from the School of Public Health, Sun Yat-sen University and Bao'an Women and Children's Hospital with approval numbers 2019-147 and LLSC-2021-02-7-06-KS, respectively.

Patients with clinical sepsis who were screened for meningitis with a lumbar puncture for CSF examination were eligible. Clinical sepsis was defined as ≥ 2 of the following: elevated or decreased counts of white blood cells (WBC, ≥30 in the first 3 days of life, ≥20 × 10^9^/L after 3 days after birth, or <5 × 10^9^/L on any day); decreased platelet count (<100 × 10^9^/L); elevated ratio of immature neutrophils to total neutrophils (I/T, ≥0.16 in the first 3 days of life, ≥0.12 after 3 days after birth); elevated level of C-reactive protein (CRP, ≥10 mg/L); and elevated level of procalcitonin (PCT, ≥0.5 mg/L) (Guida et al., 2003; Hofer et al., 2012; Hornik et al., 2012; Murphy and Weiner, 2012; Wacker et al., 2013; Wang et al., 2017). CSF samples left after relevant hematological and biochemical analyses were centrifuged at 400 × *g* at 4°C and stored at −20°C for proteomic analysis. CSF samples with a volume <0.2 mL, contaminated with blood, or centrifuged beyond 12 h after collection were discarded. Anonymized clinical information of the included infants, such as demographics and results from hematological and biochemical tests of blood closest to the CSF sampling and CSF, was also collected from the electronic medical system of the hospital.

Patients with ≥1 of the following: WBC count >20 × 10^6^/L; glucose concentration <2.2 mmol/L; or protein concentration >1,700 mg/L (Chadwick et al., 2011) were classified as the neuroinflammatory group (INF), and patients with none of the above conditions were classified as the no neuroinflammation group (NOINF).

### CSF Proteomics

CSF samples were processed according to the enhanced filter-aided sample preparation (eFASP) protocol (Erde et al., 2014). Briefly, the protein concentration was determined by a BCA protein quantification kit (Thermo Scientific, Waltham, MA, USA). CSF containing 100 μg protein was ultra-filtrated with a centrifugal filter unit (Amicon Ultra, 10 kDa, Millipore, Darmstadt, Germany) and mixed with a buffer containing sodium deoxycholate (5%) and triethylammonium bicarbonate (50 mmol/L, pH 8.0). The protein was reduced by Tris(2-carboxyethyl) phosphine (0.5 mol/L, 1:50 [v/v]), alkylated by chloroacetamide [0.5 mol/L, 1:10 (v/v)] and digested by trypsin (1 μg/100 μg protein, Promega, Madison, WI, USA, 37°C overnight) inside the spin filter with a centrifuge step (14,000 × *g* for 15 min) in between. Tryptic peptides were recovered and purified by phase extraction using ethyl acetate acidified by formic acid (1%, v/v). Vacuum-dried tryptic peptides were resuspended in 2% acetonitrile with 0.1% formic acid and applied to a Dionex RSLC UPLC System (Thermo Scientific) coupled to an Orbitrap Fusion Lumos Mass Spectrometer (Thermo Scientific). One microgram of peptides was injected onto a C18 trapping column (PepMap, 100 Å, 75 μm × 2 cm) and separated on an analytical column (Acclaim PepMap, 75 μm ID, 15 cm, 100 Å, Thermo Scientific). The peptides were eluted at a stable flow rate of 300 nL/min with a linear gradient from a solution (2.4% acetonitrile and 0.1% formic acid) to another solution (78% acetonitrile and 0.1% formic acid) in 90 min. Mass spectrometric data were obtained in the positive ionization mode in the data-dependent acquisition fashion. The mass spectra are available at the repository, proteomexchange, with the dataset identifier PXD028442.

Protein annotation and quantification were carried out using MaxQuant (version 1.5.2.8) (Cox and Mann, 2008) against the UniProt database (*Homo sapiens*, UP000005640, last modified 2021-03-07) with the detection of at least two unique peptides per protein. Processed files were imported into Perseus software (version 1.6.5.0) (Tyanova et al., 2016) to remove proteins present in <70% of the samples, common contaminants and decoys. Data containing protein identity, accession number and abundance were exported into R (version 4.0.2) (R Core Team, 2013) integrated with R Studio (RStudio Team, 2012) for further analysis.

### Data Analysis

Demographic data of the included patients were compared between the INF and NOINF groups using Student's *t*-test for normally distributed continuous variables, Wilcoxon rank sum test for continuous non-parametric variables, and Chi-square test for categorical variables.

For the analysis of the proteomic data, the abundance of proteins in each sample was divided by the total protein concentration determined by a biochemical assay to be converted into quasi-concentrations and was 2-based logarithm transformed. A score plot of principal component analysis (PCA) was prepared to assess the overall difference between the two groups using the “pcaMethods” package. To search for proteins with differential abundance between the INF and NOINF patients, a linear model was fitted to each protein with gestational age (GA) and sex as covariates. The *P*-value for assessing the significance of the difference between different neuroinflammation statuses (INF vs. NOINF) was obtained by the F test. *P*-values for all proteins were further adjusted by the false discovery rate (FDR) into *q*-values. Proteins with *q* < 0.05 and difference in mean between groups larger than one (this equals to a 2-fold change in raw abundance) were selected as proteins with different abundance.

These proteins were applied to overrepresentation (OR) analysis to be assigned to different pathways against the GO database “org.Hs.eg.db” (Carlson, 2019) using the package “clusterProfiler” (Wu et al., 2021). In addition, the abundance of selected proteins was applied to K-nearest neighbor (KNN) clustering using the package “cluster” (Maechler et al., 2021) after the missing values were imputed with the probabilistic PCA (PPCA) method using the package “pcaMethods”. Proteins appeared in both the enrichment and clustering analyses were grouped for functional discussion. A heatmap of these proteins was prepared using the “pheatmap” package based on the *Z*-scores (the standard deviations from the mean of all samples) of the logarithm-transformed abundance of proteins with missing values imputed with PPCA. On the heatmap, all samples were clustered by Hierarchical Clustering based on Euclidian distance. To search for potential correlations, proteomic and clinical data (hematological and blood biochemical data) were subjected to Spearman correlation analysis in pairwise fashion with *P*-values corrected by FDR. Correlations with FDR-adjusted *P* < 0.05 and correlation coefficient > 0.60 were selected for discussion.

## Results

During the study period (January to June 2021), a total of 46 patients were screened, and 10 samples were discarded due to contamination by hemolysis or delayed centrifugation (>12 h). Nine samples from patients showing signs of neuroinflammation were included as the INF group, while other 20 were selected from the samples left and included as the NOINF group. The demographic, hematological and biochemical data of the included patients are shown in Table 1. No significant difference was found in sex (male 13/20 vs. 5/9, NOINF vs. INF), GA [median 39.0 (IQR: 38.1–39.9) vs. 38.1 (29.4–39.1)] or birthweight [median 3,045.0 (IQR: 2,527.5–3,355.0) vs. 3,040.0 (950.0–3,500.0)] between the two groups (Ps > 0.05). Two cases of intraventricular hemorrhage (IVH) were found in each group, and no case of periventricular leukomalacia (PVL) was observed in the study population. Seizures were observed in three INF patients and none in the NOINF group. One case of bilirubin encephalopathy was found in the INF group. Only two INF and one NOINF patients showed positive result of blood culture, and except for three cases in the NOINF groups all patients received antibiotic treatment. With these low case numbers, no statistical test was conducted due to the limited power. No significant difference was found in the time from birth to CSF sampling between the two groups (*P* > 0.05, two-tailed *t*-test). No significant difference was found in the biochemical and hematological parameters in CSF and blood between the two groups, except for the total protein in the CSF (Table 1).

**Table 1:** Basic characteristics of the infants included and their hematological and biochemical readouts.

**Outcomes**	**NOINF (*n* = 20)**	**INF (*n* = 9)**	***P***
Male sex, *n* (%)	13 (65.0)	5 (55.5)	0.69^a^
GA, weeks, median (IQR)	39.0 (38.1–39.9)	38.1 (29.4–39.1)	0.19^b^
Birthweight, g, median (IQR)	3,045.0 (2,527.5–3,355.0)	3,040.0 (950.0–3,500.0)	0.83^b^
Days from birth to CSF sampling, d, median (IQR)	9.0 (1.0–24.7)	7.0 (4.0–17.0)	0.72^b^
Days from birth to blood sampling, d, median (IQR)	8.0 (1.0–24.5)	7.0 (3.0–17.0)	0.58^b^
Duration of antibiotic use before CSF sampling, d, median (IQR)	1.0 (1.0–7.5)	2.0 (2.0–3.0)	<0.01^b^
**CSF and blood biochemical parameters (mean** **±SD)**
CSF monocyte count, × 10^6^/L	3.1 ± 2.0	23.0 ± 49.5	0.26^c^
CSF chloride, mmol/L	117.0 ± 3.9	118.1 ± 5.6	0.60^c^
CSF WBC count, × 10^6^/L	3.5 ± 1.9	25.7 ± 54.9	0.26^c^
CSF glucose, mmol/L	3.0 ± 0.5	3.4 ± 1.8	0.53^c^
CSF protein, mg/L	768.9 ± 228.0	1,839.1 ± 1,247.0	0.03^c^
Blood WBC count, × 10^9^/L	13.7 ± 6.7	18.2 ± 17.2	0.46^c^
Blood neutrophil, %	62.1 ± 16.3	62.3 ± 19.9	0.98^c^
Blood lymphocyte, %	25.6 ± 13.0	25.7 ± 15.9	0.98^c^
Blood monocyte, %	14.6 ± 21.6	9.9 ± 5.7	0.38^c^
Blood platelet count, × 10^9^/L	264.6 ± 139.3	220.9 ± 108.0	0.37^c^
Blood I/T (*n* = 14 vs. *n* = 6)	16.6 ± 9.6	10.3 ± 5.0	0.07^c^
Blood CRP, mg/L	49.0 ± 45.8	32.5 ± 44.8	0.37^c^
Blood PCT, mg/L (*n* = 18 vs. *n* = 7)	20.8 ± 33.1	14.1 ± 14.4	0.49^c^

a *Tested with Fisher's test*.

b *Tested with Wilcoxon rank sum test*.

c *Tested with Student's t-test*.

**Figure 1:**
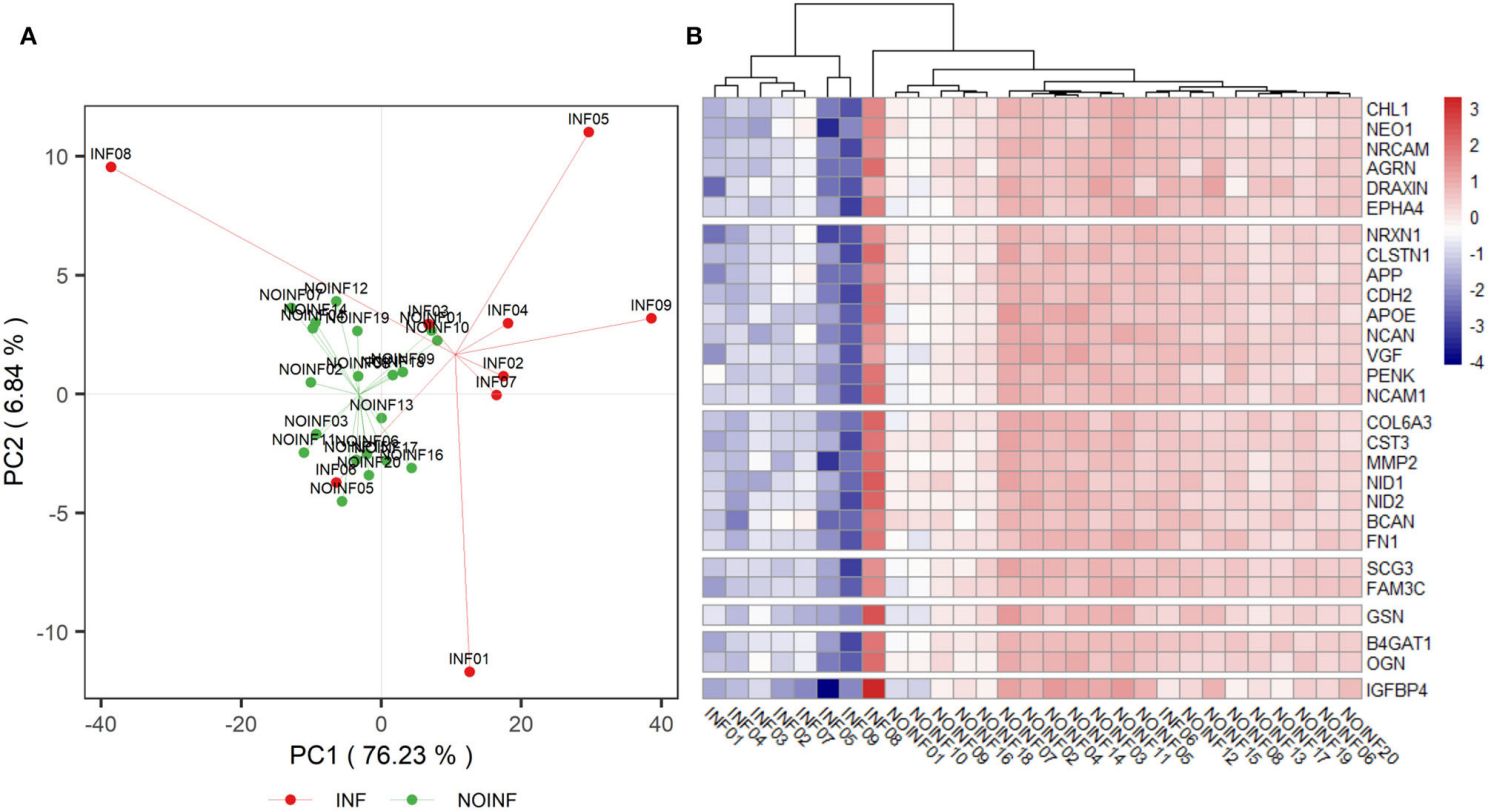
PCA score plot of detected proteins **(A)** and a heatmap of proteins listed in Table 2 **(B)**. A trend of separation between the NOINF and INF groups and a large variation in the INF group were observed in the PCA score plot. All proteins listed in Table 2 were plotted in the heatmap based on the *z*-scores of their logarithm-transformed abundance and all patients were clustered with Hierarchical Clustering based on Euclidian distance.

In total, 1,150 proteins in the CSF samples were annotated with identities, and 226 CSF proteins meeting the criteria listed above were included in the abundance analysis. A PCA score plot is shown in Figure 1A. A separation was found between the INF and NOINF patients, and a larger variation in the INF patients was also observed. Sixty-five proteins were determined to have different abundances (*q* < 0.05 and difference in logarithm-transformed abundance >1, i.e., 2-fold changes in the raw values), with all of them showing lower abundance in the INF group. Information on these proteins, including UniProt accession number, gene name, protein name, and abundance in each group, is listed in Supplementary Table S1.

An enrichment plot based on the proteins with different abundance is shown in Supplementary Figure S1, showing the first eight enriched pathways, including extracellular structure organization, axon guidance, regulation of synapse organization, platelet degranulation, receptor clustering, amyloid fibril formation and negatively regulation of long-term synaptic potentiation. Twelve clusters were formed in the clustering analysis. In total, 28 proteins were found in both the clustering and enrichment analyses and are listed in Table 2. For the purpose of clearer discussion, they were further classified into six functional groups, including axon guidance/extension, synapse organization/synaptic transmission, extracellular matrix (ECM), platelet degranulation, amyloid fiber formation, carbohydrate metabolism and regulation of the wnt pathway. These functional groups were set as mutually exclusive for easier grouping and discussion, but the functions of these proteins could be multifaceted. A heatmap of these proteins is shown in Figure 1B. The patient “INF08” from the INF group was different from the other INF patients, shown in both the PCA score plot and the heatmap. The patient “INF06” was clustered with the NOINF patients, whereas the patient “INF03”, though close to the NOINF patients in the PCA plot, was clustered with other INF patients in the heatmap.

**Table 2:** Selected proteins with differential abundance between the INF and NOINF groups.

**Accession number**	**Gene name**	**Protein name**	**NOINF^a^**	**INF^a^**	***q* ^b^**	**Specific function**
**Axon guidance/extension**						
O00533	CHL1	Neural cell adhesion molecule L1-like protein	20.29 ± 0.61	18.34 ± 2.07	<0.01	Neurite outgrowth, suppress neuronal death
Q92859	NEO1	Neogenin	18.03 ± 0.47	16.33 ± 2.29	0.01	Adhesion, neuron migration
Q92823	NRCAM	Neuronal cell adhesion molecule	19.67 ± 0.64	17.49 ± 2.30	<0.01	Axonogenesis, neuron migration & differentiation
O00468	AGRN	Agrin	19.43 ± 0.57	17.48 ± 2.26	0.01	Neurite outgrowth, synapse organization
Q8NBI3	DRAXIN	Draxin	17.37 ± 0.57	15.56 ± 1.60	<0.01	Forebrain development, suppress neuronal death
P54764	EPHA4	Ephrin type-A receptor 4	19.40 ± 0.80	17.29 ± 2.45	0.01	Axon guidance, adhesion
**Synapse organization/synaptic transmission**						
Q9ULB1	NRXN1	Neurexin-1	17.91 ± 0.54	15.53 ± 2.82	<0.01	Synaptic assembly, specification and maturation
O94985	CLSTN1	Calsyntenin-1	19.71 ± 0.58	17.88 ± 2.21	0.01	Synaptic assembly, transmission, transport to synapse
P05067	APP	Amyloid-beta precursor protein	19.45 ± 0.73	16.93 ± 3.03	0.01	Synaptogenesis
P19022	CDH2	Cadherin-2	19.98 ± 0.44	18.21 ± 2.00	<0.01	Synaptic vesicle clustering, cerebral cortex development
P02649	APOE	Apolipoprotein E	23.40 ± 0.71	21.68 ± 1.90	0.01	AMPA glutamate receptor clustering
O14594	NCAN	Neurocan core protein	18.71 ± 0.67	16.42 ± 2.24	<0.01	Cell adhesion, brain development
O15240	VGF	Neurosecretory protein VGF	19.19 ± 0.79	17.04 ± 1.94	<0.01	Regulation synaptic plasticity, memory
P01210	PENK	Proenkephalin-A	18.92 ± 0.63	17.13 ± 2.22	0.01	Synaptic transmission
A0A087WTF6	NCAM1	Neural cell adhesion molecule 1	19.48 ± 0.71	17.71 ± 2.23	0.01	Neuron-neuron adhesion, neurite outgrowth
**ECM**
P12111	COL6A3	Collagen α-3 (VI) chain	18.33 ± 0.54	16.69 ± 1.88	0.01	Cell adhesion, amyloid beta binding
P01034	CST3	Cystatin-C	25.89 ± 0.52	23.86 ± 2.16	<0.01	Neuroprotection, inhibitor of cysteine proteinases
P08253	MMP2	72 kDa type IV collagenase	18.93 ± 0.48	17.53 ± 2.04	0.02	Migration, neuroinflammation
P14543	NID1	Nidogen-1	17.94 ± 0.52	16.24 ± 2.12	0.01	Cell-substrate adhesion
Q14112	NID2	Nidogen-2	17.48 ± 0.46	16.03 ± 1.92	0.02	Cell-substrate adhesion
Q96GW7	BCAN	Brevican	20.56 ± 0.53	18.19 ± 2.89	0.01	Cell adhesion, CNS development
P02751	FN1	Fibronectin	20.72 ± 0.63	19.06 ± 1.95	0.01	Cell adhesion, axon extension
**Platelet degranulation**						
Q8WXD2	SCG3	Secretogranin-3	20.03 ± 0.69	17.84 ± 2.19	<0.01	Angiogenesis, astrocyte activation
Q92520	FAM3C	Protein FAM3C	22.44 ± 0.70	20.30 ± 2.04	<0.01	Development
**Amyloid fibril formation**						
P06396	GSN	Gelsolin	20.81 ± 0.71	19.35 ± 1.85	0.02	CNS development, anti-apoptosis
**Carbohydrate metabolism**						
O43505	B4GAT1	β-1,4-glucuronyltransferase 1	20.15 ± 0.63	18.04 ± 2.52	0.01	Axon guidance
P20774	OGN	Mimecan	20.15 ± 0.54	18.56 ± 1.91	0.01	Growth factor activity
**Regulation of wnt pathway**						
P22692	IGFBP4	Insulin-like growth factor-binding protein 4	19.08 ± 0.53	17.68 ± 0.70	<0.01	Regulation of cell growth

a *The abundance of proteins in each sample was divided by the total protein concentration determined by a biochemical assay to be converted into quasi-concentrations and was 2-based logarithm transformed*.

b *FDR-adjusted P-values*.

**Figure 2:**
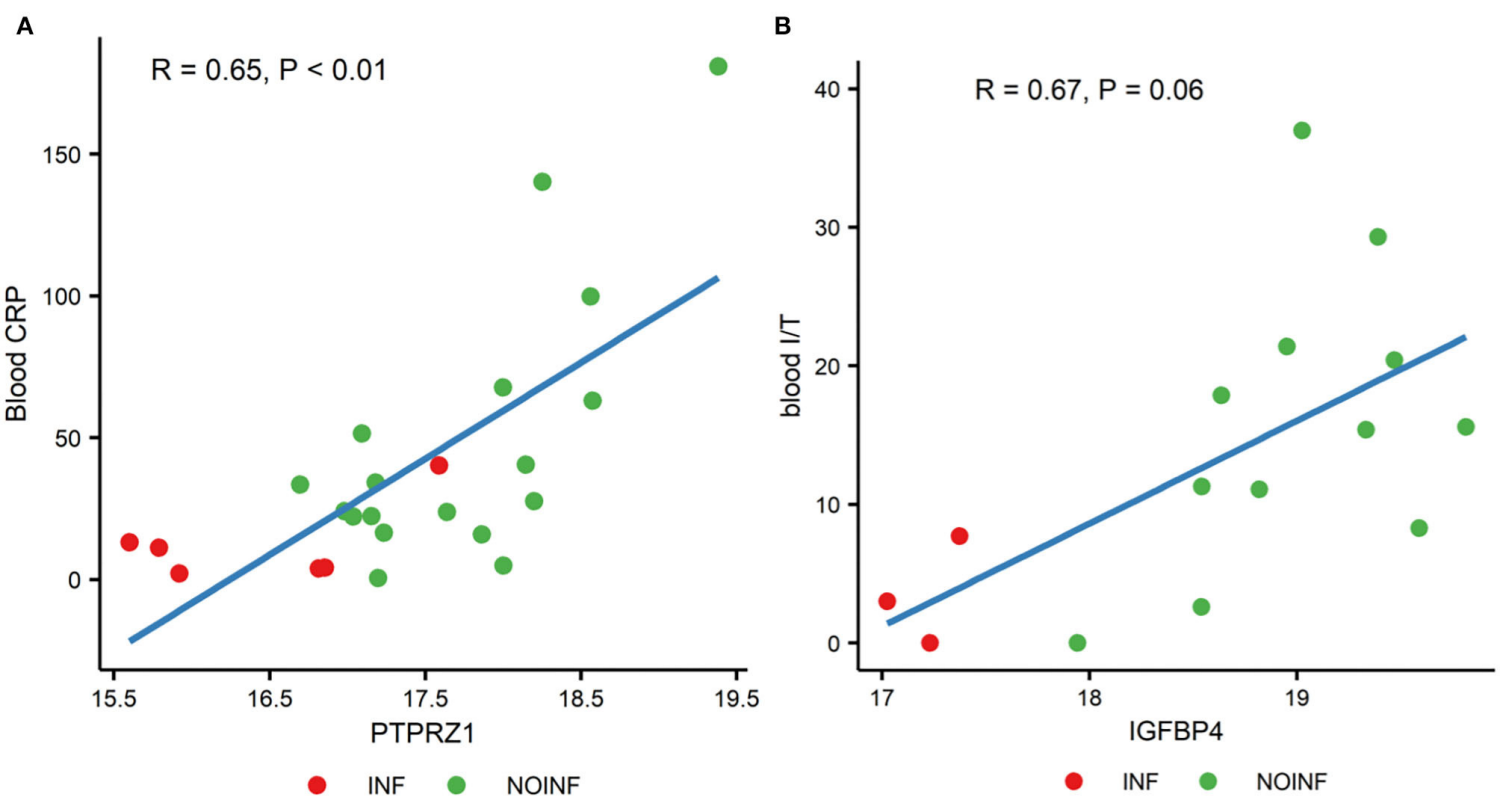
Correlations between the levels of PTPRZ1 and blood CRP **(A)** and the levels of IGFBP4 and the ratios of blood immature and total neutrophils [blood I/T, **(B)**].

Out of all possible correlations of the proteins with differential abundance and blood parameters, only two reached statistical significance even after the *P*-value correction for multiple testing by FDR. The CSF levels of protein tyrosine phosphatase receptor type Z1 (PTPRZ1) were correlated with the blood CRP levels (*P* < 0.01, correlation coefficient *R* = 0.65), and the CSF levels of insulin-like growth factor-binding protein 4 (IGFBP4) were with a trend of being correlated with the ratios of immature to total neutrophils in blood (*P* = 0.06, *R* = 0.67). Scatter plots of these two correlations are shown in Figure 2.

## Discussion

In this study, neonates with clinical sepsis and screened for meningitis were included and classified by the status of neuroinflammation. The two groups had similar demographic data and septic conditions but essentially different CSF proteomes. The CSF proteins with differential abundance affected by neuroinflammation may play a role in sepsis-related brain developmental predisposition to long-term neuropsychiatric and neurodegenerative conditions.

The perinatal period is characterized by active synaptogenesis, and studies have associated neuroinflammation in this period with adverse outcomes in later life, such as ASD, ADHD and schizophrenia (Hagberg et al., 2012). In this study, not only a difference between the INF and NOINF patients, but also a much more diverse protein profile were observed in the INF patients, despite similar demographics and hematology and biochemistry of the two groups. Of note, the INF patients clustered with the NOINF patients in the heatmap were diagnosis only by low level of CSF glucose. This suggests a diverse CSF protein profile among (clinically) neuroinflammatory patients, which may be suggestive of possible subclasses of neuroinflammation under current diagnosis criteria. Whether the patients diagnosed by different criteria as neuroinflammation have different trajectories of brain development warrants a follow-up study on a larger number of patients. A limited number of cases of epilepsy, IVH, and bilirubin encephalopathy were also found in the INF patients. As neuroinflammation is a part of the pathogenesis of these disorders (Watchko, 2016; Rana and Musto, 2018; Jiang et al., 2019), they may also have contributed to the neuroinflammation observed.

The proteins with differential abundance between the INF and NOINF patients are involved in various aspects of development and maturation of the nervous system, including synapto- and axonogenesis, neurite growth, cell adhesion and synaptic transmission, and were found at a lower abundance in the neuroinflammatory group, indicating the inflammation-related disturbance of neuronal circuit formation in these patients. Among these proteins, amyloid-β precursor protein (APP) is associated with various aspects of neurodevelopment, such as neurite growth, synaptogenesis, synaptic plasticity, neuronal adhesion and axonogenesis (Baumkötter et al., 2014). In preterm infants with posthemorrhagic hydrocephalus, a complication of IVH, the CSF levels of soluble APP are associated with ventricular sizes (Morales et al., 2015), suggesting a close relationship between CSF APP levels and cerebral injuries. In the brain tissue of infants with PVL, APP expression was colocalized with axons around the necrotic foci (Deguchi et al., 1999). The CSF levels of amyloid-β (Aβ) peptide, an APP cleaved product, are found at lower levels in adult with tuberculous meningitis (Stroffolini et al., 2021), supporting its association with meningitis. Of note, Aβ fibrils can entrap and destroy bacterial pathogens, thus Aβ is suggested as an antimicrobial peptide (Gosztyla et al., 2018). Aβ directly interacts with ephrin type-A receptor 4 (EPHA4) and agrin, which are involved in axon guidance and synaptic plasticity (Ksiazek et al., 2007) and were less abundant in the INF group. APP is functionally related to cadherin-2 (CDH2), which enhances APP dimerization (Asada-Utsugi et al., 2011), and calsyntenin-1 (CLSTN1), which mediates APP axonal transport (Vagnoni et al., 2012). Unlike our findings, the CSF levels of ApoE increased in bacterial meningitis in pediatric patients aged from 2 months to 13 years (Wang et al., 2012). In this study, the CSF levels of APP were also found to be correlated with those of NCAM1, which is abundantly expressed in the CNS and centrally involved in synaptic connectivity and cortical circuit formation (Morales et al., 2015). In line with our findings, inflammatory conditions decreased the expression of NCAM1 in the hippocampus and other brain regions of rats exposed to experimental autoimmune encephalomyelitis (Jovanova-Nesic and Shoenfeld, 2006). In addition to its neuronal expression, APP is synthesized in peripheral organs; thus, the soluble APP in CSF could, at least in part, be from the peripheral blood, given that the integrity of the BBB is compromised during infection and inflammation with increased permeability to proteins (Yap and Perlman, 2020).

Other identified proteins control different steps of neurite and/or axonal growth and synaptogenesis, including neurexin-1 (NRXN1), neuronal cell adhesion molecule (NRCAM), cell adhesion molecule L1 like (CHL1) and neuronal cadherin (CDH2). The expression of a presynaptic cell adhesion molecule, NRXN1, is disrupted in ASD, schizophrenia and intellectual disability (Ching et al., 2010; Wang et al., 2012). The alternatively spliced NRXNs are known to differentially cluster specific ligands in the postsynaptic membrane and inflammation modulates the splicing of these key presynaptic proteins (Marchese et al., 2021). Deletion of the *Chl1* gene in mice decreases the number of inhibitory interneurons and impairs GABAergic synaptic plasticity (Schmalbach et al., 2015). CDH2 and NrCAM are involved in neuronal migration (László et al., 2020) and neuron-glia interactions (Mohan et al., 2019), respectively, and are related to neuropsychiatric disorders, including ASD (Sakurai et al., 2006) and synaptic degeneration, such as AD (Hu et al., 2010). Draxin and neogenin (NEO1) are involved in thalamocortical development (Shinmyo et al., 2015). Plasma level of axonal guidance protein NEO1 is a predictor marker of survival of critically ill pediatric patients following the inflammation (Schlegel et al., 2018). Decreased abundance of these proteins in the CSF suggests that septic conditions compromise the formation of the synaptic network and may predispose to the development of a spectrum of neuropsychiatric disorders, especially those associated with synaptic malformation and function (Hagberg et al., 2012).

In the developing brain, microglia controls synaptogenesis (Wlodarczyk et al., 2017; Wilton et al., 2019) and myelination (Wlodarczyk et al., 2017), and its altered activation in response to septic conditions may directly and indirectly *via* production of cytokines and growth factors interferes with the neuronal circuits formation (Bilbo and Schwarz, 2012). VGF, a neurosecretory protein, is a precursor of a number of cleaved peptides, including TLQP-21, which can promote anti-inflammatory phenotype of microglia (Elmadany et al., 2020), thus the decreased abundance of VGF in the INF group may delay the anti-inflammatory microglial response in the developing brain. Besides, decreased levels of EPHA4 may facilitate an anti-inflammatory microglia phenotype, since *EphA4* deletion inhibits microglia proliferation and promotes the M2 polarization (Wei et al., 2019). Preclinical studies in pigs showed that the microglial response to inflammatory challenges is transient (Muk et al., 2022), however the early-life immune activation makes this long-living tissue resident macrophages more sensitive to a secondary inflammatory insult later in life, and may also have long-term behavior impact (Bilbo et al., 2006).

Another large group of identified proteins are related to the extracellular matrix (ECM), which provides a growth-permissive microenvironment and affects various aspects of brain function and development (Gaudet and Popovich, 2014). Although the exact roles of neuronal ECM in inflammation-affected neurodevelopment are not yet fully understood, multiple proteins related to the ECM have been associated with neuroinflammation. Among the proteins detected in this study, 72 kDa type IV collagenase (MMP2) is an ECM-degrading enzyme. It supports the stability and plasticity of synaptic circuits under physiological condition (Akol et al., 2022) and is involved in proteolytic degradation of basement membrane constituents in inflammation to allow entry of peripheral monocytes and macrophages into the brain (Leonardo and Pennypacker, 2009). Thus, the lower levels of MMP2 in neuroinflammation seem to be neuroprotective. Cystatin-C (CST3), an inhibitor of proteases, protects the CNS from bacterial and endogenous cysteine proteases. In line with our observation, the cystatin-C CSF levels are significantly decreased in patients with inflammatory neurological diseases (Nagai, 2008). Brevican, a CNS-specific lectican, stabilizes the composition of the ECM and plays roles in neurite outgrowth and synaptogenesis (Ajmo et al., 2008). CSF levels of brevican and APP decrease post cranial radiation following the clinical situation with cognitive decline and memory dysfunction (Fernström et al., 2018), suggesting a negative effect on the ECM and plasticity. Nidogen-1 and−2 and fibronectin 1 are essential components of the basement membranes that are important for cell movement through the ECM (Fernström et al., 2018). The CSF levels of Nidogen-2 are lower in pigs infected by bacteria than in the controls (Muk et al., 2019). Similar to the findings in various other inflammation-involving CNS pathologies, the lower CSF levels of the proteins detected here suggest an active destruction of the ECM in response to neuroinflammation. As discussed above, these proteins could be synthesized locally in the CNS, but they may also be from the peripheral tissue due to the more permeable blood brain barrier as a result of neuroinflammation.

Our results also showed that the changes in CSF proteins that could be related to systemic parameters, but probably only to a limited extend, as out of the 65 proteins with different abundance, only two showed (trend of) association with systemic parameters. PTPRZ1 is involved in axonogenesis and myelination, key processes in neonatal brain development (Tanga et al., 2019). The CSF levels of receptor-type tyrosine-protein phosphatase ζ (PTPRZ1) were positively associated with the plasma CRP levels in this study. The ratio of immature to total neutrophils (I/T) indicating neonatal sepsis was positively correlated with the CSF levels of insulin-like growth factor-binding protein 4 (IGFBP4), which regulates growth by modulating IGF function, suggesting that systemic inflammation may affect IGF activities in the CNS. Systemic levels of CRP and the ratio of immature to total neutrophils are early indicators of neonatal sepsis. These two correlations, combined, may suggest that systemic septic or inflammatory status is associated with protein changes in the CSF. However, as there is no evidence of direct interactions between PTPRZ1 and CRP or between IGFBP4 and circulating neutrophils, it could be the elevated systemic inflammation, in general, that affects the levels of CSF proteins. What need to bear in mind is that these are merely the results of correlation analysis, via which mechanism systemic inflammation affects protein levels in CSF and its potential impact on brain development of infants requires further study.

## Conclusion

In this study, proteins in CSF were profiled to identify the proteins associated with sepsis-induced neuroinflammation to provide information on inflammation-affected neurodevelopment in neonates. Neuroinflammation is associated with a decreased abundance of a broad group of proteins involved in axonal and synaptic network development and ECM homeostasis. Changes in the abundance of certain proteins are associated with systemic factors. The results suggest that neuroinflammation may impact neural circuit formation and thus may be involved in the early predisposition of brain development to neuropsychiatric disorders and neurodegenerative diseases. However, the roles of these proteins in inflammation-affected neurodevelopment remain largely elusive, and their value as biomarkers for the prediction of long-term morbidities remains undetermined and thus requires further investigation.

## Data Availability Statement

The datasets presented in this study can be found in online repositories. The names of the repository/repositories and accession number(s) can be found below: http://www.proteomexchange.org/, PXD028442.

## Ethics Statement

The studies involving human participants were reviewed and approved by the School of Public Health, Sun Yat-sen University and Bao'an Women and Children's Hospital. Written informed consent to participate in this study was provided by the participants' legal guardian/next of kin.

## Author Contributions

P-PJ, PL, and YC contributed to conception and design of the study. YC and PZ organized the sample collection. S-SP performed the analysis. P-PJ and PL performed the data analysis. P-PJ wrote the first draft of the manuscript. SP wrote sections of the manuscript. All authors contributed to manuscript revision, read, and approved the submitted version.

## Funding

This work was financially supported by Sun Yat-sen University with a starting grant to P-PJ.

## Conflict of Interest

The authors declare that the research was conducted in the absence of any commercial or financial relationships that could be construed as a potential conflict of interest.

## Publisher's Note

All claims expressed in this article are solely those of the authors and do not necessarily represent those of their affiliated organizations, or those of the publisher, the editors and the reviewers. Any product that may be evaluated in this article, or claim that may be made by its manufacturer, is not guaranteed or endorsed by the publisher.

## Abbreviations

AD: Alzheimer's disease
ADHD: attention deficit hyperactivity disorder
ASD: autism spectrum disorder
CNS: central nervous system
CRP: C-reactive protein
CSF: cerebrospinal fluid
ECM: extracellular matrix
FDR: false discovery rate
GA: gestational age
I/T: immature neutrophils to total neutrophils
IGF: insulin-like growth factor
IQR: interquartile range
KNN: K-nearest neighbor
MMP2: 72 kDa type IV collagenase
OR: over-representation analysis
PCA: principal component analysis
PCT: procalcitonin
PRRs: pattern recognition receptors
WBC: white blood cell. Please refer to Table 2 for full names of proteins.
