# Supplementary material for: Proteins Involved in Synaptic Plasticity Are Downregulated in the Cerebrospinal Fluid of Infants With Clinical Sepsis Complicated by Neuroinflammation
Source: Front Cell Neurosci. 2022 May 11;16:887212. doi: 10.3389/fncel.2022.887212 (PMC9130476; doi:10.3389/fncel.2022.887212)

Supplementary information

Proteins Involved in Synaptic Plasticity are Downregulated in the Cerebrospinal Fluid of Infants with Clinical Sepsis Complicated by Neuroinflammation

Ping-Ping Jiang, Shan-Shan Peng, Stanislava Pankratova, Ping Luo, Ping Zhou, You Chen

Table S1. All detected proteins with differential abundance between the NEUINF and CON groups.

Figure S1. Enrichment plots.

Table S1. All detected proteins with differential abundance between the NEUINF and Con groups.

Accession number	Protein name	Gene name	CON[a]	NEUINF[a]	*q*
A0A087WTF6	Neural cell adhesion molecule 1	NCAM1	19.48 ± 0.71	17.71 ± 2.23	0.01
Q9BY67	Cell adhesion molecule 1	CADM1	17.95 ± 0.66	16.58 ± 1.03	0.01
Q96B86	Repulsive guidance molecule A	RGMA	17.52 ± 0.52	15.90 ± 1.54	< 0.01
Q9NQ79	Cartilage acidic protein 1	CRTAC1	19.59 ± 0.74	17.36 ± 2.37	< 0.01
Q9ULB1	Neurexin-1	NRXN1	17.91 ± 0.54	15.53 ± 2.82	< 0.01
P13987	CD59 glycoprotein	CD59	18.74 ± 0.89	17.05 ± 1.12	< 0.01
P23471	Receptor-type tyrosine-protein phosphatase ς	PTPRZ1	17.73 ± 0.71	16.43 ± 0.78	< 0.01
O00468	Agrin	AGRN	19.43 ± 0.57	17.48 ± 2.26	0.01
Q99674	Cell growth regulator with EF hand domain protein 1	CGREF1	17.30 ± 0.74	15.75 ± 1.28	< 0.01
Q8TAG5	V-set and transmembrane domain-containing protein 2A	VSTM2A	19.73 ± 0.60	17.84 ± 1.52	< 0.01
P51693	Amyloid beta precursor like protein 1	APLP1	21.45 ± 0.51	19.55 ± 2.20	0.01
Q92823	Neuronal cell adhesion molecule	NRCAM	19.67 ± 0.64	17.49 ± 2.30	< 0.01
P12111	Collagen alpha-3(VI) chain	COL6A3	18.33 ± 0.54	16.69 ± 1.88	0.01
Q13822	Ectonucleotide pyrophosphatase/phosphodiesterase family member 2	ENPP2	19.22 ± 0.78	17.25 ± 2.59	0.02
P54764	Ephrin type-A receptor 4	EPHA4	19.40 ± 0.80	17.29 ± 2.45	0.01
Q9UBP4	Dickkopf-related protein 3	DKK3	20.31 ± 0.65	18.35 ± 2.21	0.01
Q13449	Limbic system-associated membrane protein	LSAMP	18.92 ± 0.73	17.18 ± 2.16	0.02
O00451	GDNF family receptor alpha-2	GFRA2	17.22 ± 0.51	15.93 ± 1.15	0.01
O00533	Neural cell adhesion molecule L1-like protein	CHL1	20.29 ± 0.61	18.34 ± 2.07	< 0.01
O14594	Neurocan core protein	NCAN	18.71 ± 0.67	16.42 ± 2.24	< 0.01
O15240	Neurosecretory protein VGF	VGF	19.19 ± 0.79	17.04 ± 1.94	< 0.01
O43505	Beta-1,4-glucuronyltransferase 1	B4GAT1	20.15 ± 0.63	18.04 ± 2.52	0.01
O94985	Calsyntenin-1	CLSTN1	19.71 ± 0.58	17.88 ± 2.21	0.01
P01034	Cystatin-C	CST3	25.89 ± 0.52	23.86 ± 2.16	< 0.01
P01210	Proenkephalin-A	PENK	18.92 ± 0.63	17.13 ± 2.22	0.01

P01344	Insulin-like growth factor II	IGF2	19.35 ± 0.63	17.64 ± 1.60	0.01
P01742	Immunoglobulin heavy variable 1-69	IGHV1-69	19.24 ± 0.56	17.51 ± 0.98	< 0.01
P02461	Collagen alpha-1(III) chain	COL3A1	19.50 ± 0.63	17.78 ± 2.20	0.02
P02649	Apolipoprotein E	APOE	23.40 ± 0.71	21.68 ± 1.90	0.01
P02751	Fibronectin	FN1	20.72 ± 0.63	19.06 ± 1.95	0.01
P05060	Secretogranin-1	CHGB	20.81 ± 1.00	18.41 ± 2.50	< 0.01
P05067	Amyloid-beta precursor protein	APP	19.45 ± 0.73	16.93 ± 3.03	0.01
P05408	Neuroendocrine protein 7B2	SCG5	19.75 ± 0.63	18.37 ± 1.69	0.01
P05452	Tetranectin	CLEC3B	20.93 ± 0.55	19.48 ± 1.90	0.02
P06396	Gelsolin	GSN	20.81 ± 0.71	19.35 ± 1.85	0.02
P08253	72 kDa type IV collagenase	MMP2	18.93 ± 0.48	17.53 ± 2.04	0.02
P09486	SPARC	SPARC	18.65 ± 0.68	16.59 ± 2.48	0.01
P10645	Chromogranin-A	CHGA	20.55 ± 0.77	18.36 ± 2.29	< 0.01
P13521	Secretogranin-2	SCG2	18.75 ± 0.75	16.25 ± 2.29	< 0.01
P14543	Nidogen-1	NID1	17.94 ± 0.52	16.24 ± 2.12	0.01
P18065	Insulin-like growth factor-binding protein 2	IGFBP2	19.69 ± 0.54	18.11 ± 1.98	0.02
P19022	Cadherin-2	CDH2	19.98 ± 0.44	18.21 ± 2.00	< 0.01
P20774	Mimecan	OGN	20.15 ± 0.54	18.56 ± 1.91	0.01
P22692	Insulin-like growth factor-binding protein 4	IGFBP4	19.08 ± 0.53	17.68 ± 0.70	< 0.01
P41222	Prostaglandin-H2 D-isomerase	PTGDS	26.55 ± 0.55	25.00 ± 2.03	0.02
P54289	Voltage-dependent calcium channel subunit α-2/δ-1	CACNA2D1	18.07 ± 0.82	15.18 ± 2.94	< 0.01
P55290	Cadherin-13	CDH13	20.39 ± 0.69	18.81 ± 1.97	0.01
P61626	Lysozyme C	LYZ	19.15 ± 0.53	18.02 ± 1.17	0.01
P61769	β-2-microglobulin	B2M	23.68 ± 0.80	22.08 ± 1.73	0.01
P98160	heparan sulfate proteoglycan core protein	HSPG2	19.32 ± 0.60	17.73 ± 1.96	0.01
Q12860	Contactin-1	CNTN1	19.04 ± 0.82	17.01 ± 2.21	0.01
Q14112	Nidogen-2	NID2	17.48 ± 0.46	16.03 ± 1.92	0.02
Q14515	SPARC-like protein 1	SPARCL1	20.06 ± 0.70	18.05 ± 2.59	0.01
Q15113	Procollagen C-endopeptidase enhancer 1	PCOLCE	19.52 ± 0.62	17.73 ± 1.65	< 0.01

Q53EL9	Seizure protein 6 homolog	SEZ6	19.17 ± 0.73	17.41 ± 2.12	0.02
Q6EMK4	Vasorin	VASN	17.09 ± 0.43	15.82 ± 0.80	< 0.01
Q6UX71	Plexin domain-containing protein 2	PLXDC2	17.64 ± 0.65	15.74 ± 1.96	0.01
Q8NBI3	Draxin	DRAXIN	17.37 ± 0.57	15.56 ± 1.60	< 0.01
Q8WXD2	Secretogranin-3	SCG3	20.03 ± 0.69	17.84 ± 2.19	< 0.01
Q92520	Protein FAM3C	FAM3C	22.44 ± 0.70	20.30 ± 2.04	< 0.01
Q92859	Neogenin	NEO1	18.03 ± 0.47	16.33 ± 2.29	0.01
Q92876	Kallikrein-6	KLK6	18.97 ± 0.94	16.91 ± 2.76	0.01
Q96GW7	Brevican core protein	BCAN	20.56 ± 0.53	18.19 ± 2.89	0.01
Q99435	Protein kinase C-binding protein NELL2	NELL2	20.91 ± 0.54	18.97 ± 2.15	< 0.01
Q9UHG2	ProSAAS	PCSK1N	20.96 ± 0.71	19.33 ± 1.93	0.01

[a]Data are 2-based logarithm transformed and shown as mean ± SD; [b] q values are FDR-adjusted p values calcualted from linear model.

Figure S1. Enricment plot of proteins with differential abundance between the NEUINF and CON infants

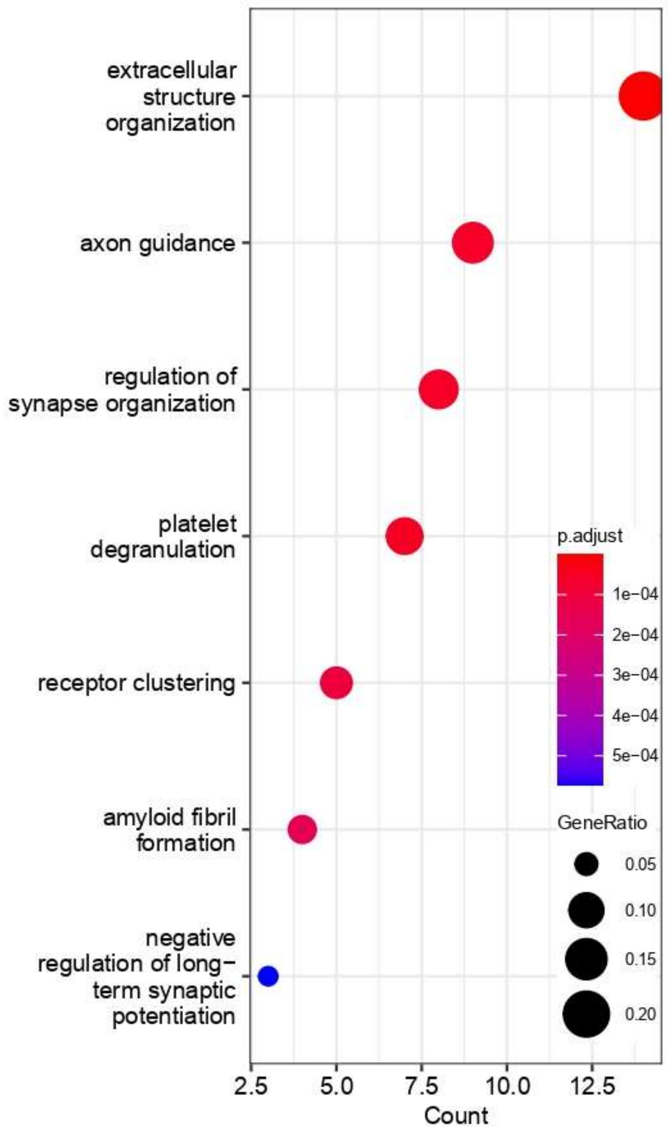

Supplement: Supplementary file 3 [file Data_Sheet_1.PDF]
